# Unemployment, public–sector health care expenditure and HIV mortality: An analysis of 74 countries, 1981–2009

**DOI:** 10.7189/jogh.05.010403

**Published:** 2015-06

**Authors:** Mahiben Maruthappu, Charlie Da Zhou, Callum Williams, Thomas Zeltner, Rifat Atun

**Affiliations:** 1 Imperial College London, London, UK; 2 Faculty of Arts and Sciences, Harvard University, Cambridge, MA, USA; 3 New College, University of Oxford, Oxford, UK; 4 The Economist, London, UK; 5 Faculty of History, University of Oxford, Oxford, UK; 6 Special Envoy for Financing to the Director General of the World Health Organization, Geneva, Switzerland; 7 Harvard School of Public Health, Boston, MA, USA

## Abstract

**Background:**

The global economic downturn has been associated with increased unemployment and reduced public–sector expenditure on health care (PSEH). We determined the association between unemployment, PSEH and HIV mortality.

**Methods:**

Data were obtained from the World Bank and the World Health Organisation (1981–2009). Multivariate regression analysis was implemented, controlling for country–specific demographics and infrastructure. Time–lag analyses and robustness–checks were performed.

**Findings:**

Data were available for 74 countries (unemployment analysis) and 75 countries (PSEH analysis), equating to 2.19 billion and 2.22 billion people, respectively, as of 2009. A 1% increase in unemployment was associated with a significant increase in HIV mortality (men: 0.1861, 95% CI: 0.0977 to 0.2744, *P* = 0.0000, women: 0.0383, 95% CI: 0.0108 to 0.0657, *P* = 0.0064). A 1% increase in PSEH was associated with a significant decrease in HIV mortality (men: –0.5015, 95% CI: –0.7432 to –0.2598, *P* = 0.0001; women: –0.1562, 95% CI: –0.2404 to –0.0720, *P* = 0.0003). Time–lag analysis showed that significant changes in HIV mortality continued for up to 5 years following variations in both unemployment and PSEH.

**Interpretation:**

Unemployment increases were associated with significant HIV mortality increases. PSEH increases were associated with reduced HIV mortality. The facilitation of access–to–care for the unemployed and policy interventions which aim to protect PSEH could contribute to improved HIV outcomes.

The recent economic downturn has had profound effects on an international scale. Governmental responses around the world have included the introduction of radical austerity measures in an attempt to reduce budget deficits by increasing taxation while cutting back expenditure [1,2]. These fiscal policies have resulted in unprecedented rises in unemployment rates [3-5] which has coincided with a period of slower than expected growth in public–sector expenditure on health care (PSEH) [6,7]. For example, in the 33 countries of the Organisation for Economic Co–operation and Development (OECD), which collectively account for more than 1.24 billion individuals, unemployment levels rose from 5.6% to 7.6% following the recession [5]. In the United States, the recession preceded the lowest annual growth in PSEH in fifty years [7]. Concurrently, there has been a substantial slowdown in the growth of internationally sourced funding for development assistance in health [8]. Such changes have raised the question of how economic changes, both within and outside of crises, impact population health.

There are widespread concerns about the potential detriment that the current economic environment may have on global public health [9]. The adverse effects of economic crises on population health outcomes, including all–cause mortality, suicide rates and mental health, have been well described [10]. These trends have also been confirmed in the context of the current recession [11-13]. The role of unemployment, specifically, in the perpetuation of a variety of unhealthy phenomena has also been confirmed [14-19]. The contribution of PSEH on population health has been shown to be modest when compared to other variables, such as sanitation and nutrition [20, 21]. PSEH does, however, seem to disproportionately benefit the poorer substrata within populations [22]. It would follow that reduced PSEH at times of increased unemployment would serve to exacerbate negative health outcomes. Despite these findings, detailed analyses of the relationship between unemployment and PSEH with specific pathologies have, by and large, been neglected.

Human immunodeficiency virus (HIV) is the leading cause of global mortality by a single pathogenic agent, predominantly affecting young adults of working age [23]. A small number of studies have investigated the effect of financial downturns on HIV–infected individuals in single country settings, concluding that unemployment is an independent risk factor for disease progression and mortality [24, 25]. However, population–wide correlations at the multinational level, in addition to long–term trends, have yet to be determined.

In this study, we sought to evaluate the association between unemployment, PSEH, and HIV mortality in 74 and 75 countries, respectively, between 1981 and 2009 (**Table 1**). Given the current economic climate and pressures to develop appropriate policy responses, we believe this to be highly topical.

**Table 1:** Countries included in our analysis

Country	Unemployment analysis	PSEH analysis
Albania	✓	✓
Argentina	✓	✓
Armenia	✓	✓
Australia	✓	✓
Austria	✓	✓
Azerbaijan	✓	✓
Bahrain	✓	✓
Belarus	✗	✓
Belgium	✓	✓
Bosnia and Herzegovina	✓	✗
Brazil	✓	✓
Bulgaria	✓	✓
Canada	✓	✓
Chile	✓	✓
Colombia	✓	✓
Costa Rica	✓	✓
Croatia	✓	✓
Cuba	✓	✓
Cyprus	✓	✓
Czech Republic	✓	✓
Denmark	✓	✓
Ecuador	✓	✓
Egypt, Arab Rep.	✓	✓
El Salvador	✓	✓
Estonia	✓	✓
Finland	✓	✓
France	✓	✓
Georgia	✓	✓
Germany	✓	✓
Greece	✓	✓
Guatemala	✓	✓
Hong Kong SAR, China	✓	✗
Hungary	✓	✓
Ireland	✓	✓
Israel	✓	✓
Italy	✓	✓
Japan	✓	✓
Kazakhstan	✓	✓
Korea, Rep.	✓	✓
Kuwait	✓	✓
Kyrgyz Republic	✓	✓
Latvia	✓	✓
Lithuania	✓	✓
Macedonia, FYR	✓	✓
Mauritius	✓	✓
Mexico	✓	✓
Moldova	✓	✓
Montenegro	✗	✓
Netherlands	✓	✓
New Zealand	✓	✓
Norway	✓	✓
Oman	✗	✓
Panama	✓	✓
Paraguay	✓	✓
Philippines	✓	✓
Poland	✓	✓
Portugal	✓	✓
Puerto Rico	✓	✗
Qatar	✓	✓
Romania	✓	✓
Russian Federation	✓	✓
Serbia	✓	✓
Singapore	✓	✓
Slovak Republic	✓	✓
Slovenia	✓	✓
South Africa	✓	✓
Spain	✓	✓
Sri Lanka	✓	✓
Sweden	✓	✓
Switzerland	✓	✓
Thailand	✓	✓
Trinidad and Tobago	✓	✓
Ukraine	✓	✓
United Kingdom	✓	✓
United States	✓	✓
Uzbekistan	✗	✓
Uruguay	✓	✓
Venezuela, RB	✓	✓
**Total population (male)**	**1 073 281 689**	**1 087 959 917**
**Percentage of world population (male)**	**30.22%**	**30.63%**
**Total population (female)**	**1 121 036 031**	**1 135 056 068**
**Percentage of world population (female)**	**32.08%**	**32.48%**
**Total population (both sexes)**	**2 194 317 721**	**2 223 015 985**
**Percentage of world population (both sexes)**	**31.14%**	**31.55%**

PSEH – public–sector expenditure on health care

## METHODS

### Data collection

Annual national HIV mortality data, between 1981 and 2009, were obtained from the World Health Organisation’s (WHO) mortality database [26]. The quality of this data has been evaluated by the WHO [27]. Reported national death statistics were assessed by completeness, coverage and quality (**Online Supplementary Document(Online Supplementary Document)**). Completeness was defined as the proportion of all deaths that are registered in the population covered by the vital registration system for a country. Coverage is calculated by dividing the total number of deaths reported from vital registration system for a country-year by the total number of deaths estimated by the WHO for that year for the national population. No adjustments have been made to the raw data to account for under-coverage. Quality was determined by taking into account the revision of the International statistical classification of diseases and related health problems (ICD) was used for national vital registration statistics, the completeness of data and minimal use of ill-defined categories of death. Of the 78 countries included in both of our analyses, data evaluation was not performed for 2 countries: Hong Kong and Porto Rico. Of those remaining, 67 countries had achieved completeness of greater or equal to 80% and 73 achieved completeness of greater or equal to 70%. 62 countries had coverage of greater or equal to 80% and 73 had coverage greater or equal to 70%. 20 countries were determined to have high quality data, 39 medium and 17 low.

As defined by the WHO, age standardised death rates (ASDR) is the weighted average of age–specific mortality rates per 100 000, where the weights are proportional to the number of persons in each corresponding age group of the WHO standard population [28]. ASDR per 100 000 was used as the basis of our statistical analysis as it controls for differences in age distribution within populations. Socioeconomic data were obtained from the World Bank’s Development Indicators Database 2013 [29]. Unemployment was taken to be the proportion of the labour force without work but available and seeking employment, as defined by the World Bank [29]. PSEH data was measured as a percentage of national gross domestic product (GDP). It is defined by the World Bank to consist of recurrent and capital spending from government budgets, external borrowings, grants and social health insurance funds [29]. Data on 74 and 75 countries were available for the unemployment and PSEH analyses respectively (**Table 1**). As of 2012, this represented 2.19 billion and 2.22 billion people respectively.

### Statistical analysis

Multivariate regression analysis was used to assess the relationship between HIV mortality (dependent variable) and unemployment and PSEH (independent variables). To ensure that results were not driven by extreme observations for certain countries, a fixed–effects approach was used in our regression models, including dummy variables for every country in the data set. Doing this meant that our model evaluated mortality changes within individual countries while holding constant time–invariant differences between countries such as a higher predisposition to HIV, as well as political, cultural and structural differences. This conservative modelling approach made the data more comparable. The demographic structure of the selected countries was also controlled for by incorporating total population size and the proportions of the population that were aged over 65 and below 15 years into the model.

We used the Cook–Weisberg test [30] to assess for and to confirm heteroskedasticity (where sub–samples have different distributions) in the data used. Therefore, robust standard errors were included in the regression models; this allowed us to account for heterogeneity in unemployment and PSEH data due to differences in the way that countries measured unemployment rates and PSEH, along with factors such as underemployment or social programmes (for example, back–to–work initiatives or programmes that see people move from unemployment into education or training) that may otherwise have hidden or suppressed actual unemployment rates.

Due to the inclusion of several control variables (which in turn results in the loss of degrees of freedom and reduced sample size), our approach was highly conservative. This methodology has been widely used in similar health–economic studies and is regarded as a statistically robust approach [19, 31-35].

The fixed effects model used was as follows:


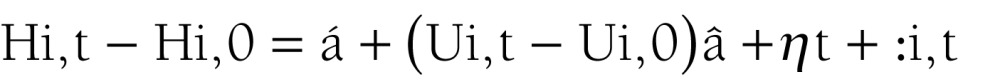


where i is country and t is year; H is the response variable or health measure (HIV mortality); U is the predictor variable (either unemployment or public–sector health care spending); α represents the population structure of the country being analysed; η is a dummy variable for each country included in the regression model; and ε is the error term. The coefficients of the control variables can be found in **Online Supplementary Document(Online Supplementary Document)**.

We conducted 1–, 2–, 3–, 4– and 5–year time–lag multivariate analyses to quantify the long–term effects of changes in unemployment and PSEH on HIV mortality. To ensure the robustness of our findings, we conducted a series of further statistical analyses on the associations of unemployment and PSEH on HIV mortality in both sexes, taking into consideration several additional control variables. First, we controlled for GDP per capita, inflation and national debt (as a percentage GDP). These markers of national economic well–being are commonly used as indicators for the standard of living and also influence national health care budgets. Second, we controlled for urbanisation, calorific intake and access to clean water. Our third robustness check combined the controls from the previous two. Fourth, we controlled for out of pocket health care expenses. Fifth, private health care expenditure (as a percentage GDP) was controlled for. Sixth, we controlled for changes in crude death rate; this accounted for mortality risk inherent to the unemployed and in countries with reduced government health care spending, allowing us to determine HIV–specific trends. Seventh, we re–ran the original multivariate regressions using data classified as either Level 1 or Level 2 in quality by the WHO. Finally, we reran the PSEH analysis with changes in PSEH measured in purchasing power parity (PPP) per capita rather than GDP. The association between both unemployment and PSEH and HIV mortality remained statistically significant (*P* < 0.05) throughout all of our robustness checks (**Table 2**).

**Table 2:** Robustness checks

Robustness check	Controls used in multiple regression	Coefficient	P value	Lower confidence interval	Upper confidence interval
Multiple regression analyses were re–run using the controls in the original analysis (population size, proportion of population above 65 y of age, proportion below 14, and individual country controls), in addition to those mentioned in the table below. A 1% rise in unemployment remains statistically associated with increased HIV mortality in both sexes across all robustness checks:					
**Economic controls**	Original analysis controls and: changes in GDP per capita, inflation and government debt as a percentage of GDP	0.0957	0.0052	0.0287	0.1627
**Infrastructure controls**	Original analysis controls and: urbanisation, access to water and nutrition (mean calorific intake)	0.1781	<0.0001	0.1086	0.2475
**Combined economic and infrastructure controls**	Original analysis controls and: changes in GDP per capita, inflation, government debt as a percentage of GDP, urbanisation, access to water and nutrition (mean calorific intake)	0.1599	0.0004	0.0715	0.2483
**Healthcare controls**	Original analysis controls and: out of pocket expenses	0.1042	0.0109	0.0241	0.1843
	Original analysis controls and: private health expenditure as a percentage of GDP	0.1108	0.0069	0.0305	0.1910
**Crude death rate controls**	Original analysis controls and: crude death rate	0.1166	<0.0001	0.0607	0.1725
**WHO data quality check**	Original analysis controls, using WHO level 1 and 2 surveillance data only	0.1218	0.0001	0.0629	0.1807
Similarly, a 1% rise in public health expenditure remains statistically associated with decreased HIV mortality in both sexes across all robustness checks:					
**Economic controls**	Original analysis controls and: changes in GDP per capita, inflation and government debt as a percentage of GDP	–0.4369	0.0002	–0.6647	–0.2091
**Infrastructure controls**	Original analysis controls and: urbanisation, access to water and nutrition (mean calorific intake)	–0.3880	0.0007	–0.6110	–0.1649
**Combined economic and infrastructure controls**	Original analysis controls and: changes in GDP per capita, inflation, government debt as a percentage of GDP, urbanisation, access to water and nutrition (mean calorific intake)	–0.4104	0.0094	–0.7191	–0.1012
**Healthcare controls**	Original analysis controls and: out of pocket expenses	–0.3270	0.0004	–0.5065	–0.1475
	Original analysis controls and: private health expenditure as a percentage of GDP	–0.3225	0.0001	–0.4804	–0.1646
**Crude death rate controls**	Original analysis controls and: crude death rate	–0.2755	0.0015	–0.4450	–0.1061
**WHO data quality check**	Original analysis controls, using WHO level 1 and 2 surveillance data only	–0.3260	0.0001	–0.4841	–0.1679
Alternative PSEH measure	Rerun original analysis with PSEH measured in PPP per capita	–0.0009	<0.0001	–0.0012	–0.0006

GDP – gross domestic pruduct, WHO – World Health Organization, PSEH – public–sector expenditure on health care, PPP – purchasing power parity

Stata SE version 12 was used for the analysis (Stata Corporation, Texas, USA).

## RESULTS

The results of our regression analyses evaluating the effects of unemployment and PSEH on HIV mortality per 100 000, 1981–2009, controlling for inter–country differences in infrastructure and demographics, are shown in **Table 3**.

**Table 3:** Multiple regression and lag analysis

Number of years after 1% rise in unemployment	Male HIV mortality per 100 000					Female HIV mortality per 100 000			
**Coefficient**	**P value**		**Lower confidence interval**	**Upper confidence interval**	**Coefficient**	**P value**	**Lower confidence interval**	**Upper confidence interval**
Impact of a hypothetical 1% rise in unemployment on HIV mortality, controlling for proportion of population under the age of 14, proportion of population over the age of 65, population size and 74 country controls:									
Year 0 (year of change in unemployment)	0.1861	<0.0001		0.0977	0.2744	0.0383	0.0064	0.0108	0.0657
Year 1	0.1523	0.0008		0.0636	0.2411	0.0345	0.0101	0.0082	0.0607
Year 2	0.1436	0.0008		0.0603	0.2270	0.0446	0.0007	0.0190	0.0702
Year 3	0.0964	0.0100		0.0231	0.1697	0.0395	0.0023	0.0141	0.0649
Year 4	0.0551	0.1421		–0.0185	0.1288	0.0352	0.0123	0.0077	0.0628
Year 5	0.0621	0.1306		–0.0184	0.1425	0.0377	0.0260	0.0045	0.0709
The impact of a 1% rise in public health expenditure on HIV mortality, controlling for proportion of population under the age of 14, proportion of population over age of 65, population size and 75 country controls:									
Year 0 (year of change in unemployment)	–0.5015	0.0001	–0.7432		–0.2598	–0.1562	0.0003	–0.2404	–0.0720
Year 1	–0.5398	0.0008	–0.8537		–0.2258	–0.2105	0.0024	–0.3460	–0.0749
Year 2	–0.4704	0.0011	–0.7518		–0.1890	–0.1623	0.0098	–0.2853	–0.0393
Year 3	–0.5063	0.0005	–0.7916		–0.2210	–0.1881	0.0022	–0.3080	–0.0682
Year 4	–0.4674	0.0026	–0.7705		–0.1642	–0.1599	0.0165	–0.2906	–0.0292
Year 5	–0.3511	0.0218	–0.6507		–0.0514	–0.0620	0.2863	–0.1760	0.0521

### Unemployment

A 1% rise in unemployment was found to be associated with a statistically significant immediate rise in HIV mortality in both males (coefficient 0.1861, 95% CI: 0.0977 to 0.2744, *P* < 0.0001) and females (coefficient 0.0383, 95% CI: 0.0108 to 0.0657, *P* = 0.0064). As of 2012, the combined populations of the 74 countries in our analysis was in excess of 2.19 billion individuals.

Lag analysis showed that unemployment rises were associated with significantly increased HIV mortality for several years following the initial change (**Table 3**). In males, there is a significant association for 3 years after the rise in unemployment. In year 1, coefficient 0.1523, 95% CI: 0.0636 to 0.2411, *P* = 0.0008. In year 2, coefficient 0.1436, 95% CI: 0.0603 to 0.2270, *P* = 0.0008. And in year 3, coefficient 0.0964, 95% CI: 0.231 to 0.1697, *P* = 0.0100. After this interval, the association becomes non–significant. In females, the association remains statistically significant for at least 5 years. In year 1, coefficient 0.0345, 95% CI: 0.0082 to 0.0607, *P* = 0.0101. In year 2, coefficient 0.0446, 95% CI: 0.0190 to 0.0702, *P* = 0.0007. In year 3, coefficient 0.0395, 95% CI: 0.0141 to 0.0649, *P* = 0.0123. In year 4, coefficient 0.0352, 95% CI: 0.0077 to 0.0628, *P* = 0.0123. And in year 5, coefficient 0.0377, 95% CI: 0.0045 to 0.0709, *P* = 0.0260.

### PSEH

The combined population of the 75 countries included in our PSEH analysis exceeded 2.22 billion individuals in 2012. A 1% rise in PSEH was found to be associated with a significant reduction in HIV mortality. Within the first year following a 1% increase in PSEH, the ASDR of HIV changed by a coefficient of –0.5015, 95% CI: –0.7432 to –0.2598, *P* = 0.0001 in males, and by a coefficient of –0.1562, 95% CI: –0.2404 to –0.0720, *P* = 0.0003 in females.

Lag analysis of the PSEH data showed that these associations with HIV mortality persisted for at least 5 years in males. For year 1, coefficient –0.5398, 95% CI: –0.8537 to –0.2258), *P* = 0.0008. In year 2, coefficient –0.4704, 95% CI: 0.7518 to –0.1890, *P* = 0.0011. In year 3, coefficient –0.5063, 95% CI: –0.7916 to –0.2210, *P* = 0.0005. In year 4, coefficient –0.4674, 95% CI: –0.7705 to –0.1642, *P* = 0.0026. And in year 5, coefficient –0.3511, 95% CI: –0.6507 to –0.0514, *P* = 0.0218. In females, the statistically significant association persisted for 4 years following the change in PSEH. For year 1, coefficient –0.2105, 95% CI: –0.3460 to –0.0749, *P* = 0.0024. In year 2, coefficient –0.1623, 95% CI: 0.2853 to –0.0393, *P* = 0.0098. In year 3, coefficient –0.1881, 95% CI: –0.3080 to –0.0682, *P* = 0.0022. In year 4, coefficient –0.1599, 95% CI: –0.2906 to –0.0292, *P* = 0.0165.

## DISCUSSION

This study demonstrates that both increased unemployment and decreased PSEH are associated with increased HIV mortality on a global scale. Changes in these two parameters have an immediate association with changes HIV mortality which continues into the medium–term. The significance of these findings persisted even after consideration of a variety of potential confounders, including demographic, economic, infrastructure, health care, and data quality related factors.

### Mechanisms

**Figure 1:**
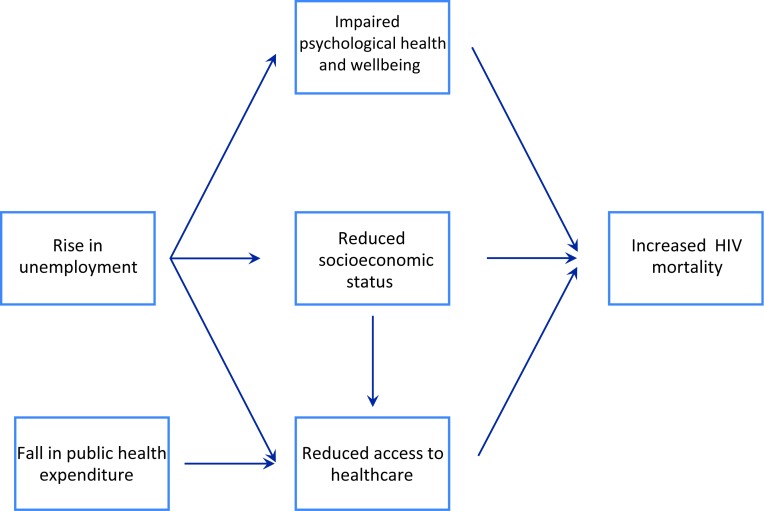
Mechanisms that may underlie a potential causal link between unemployment and HIV mortality.

We propose a number of mechanisms that may underlie a potential causal link between unemployment and HIV mortality (**Figure 1**). First, unemployment may contribute towards the reduced socioeconomic status of HIV–infected individuals. A number of studies have previously shown that low socioeconomic status is associated with an increased risk of HIV mortality [36-39]. Some have concluded that this the result of reduced health care access which in turn results in delayed diagnosis and treatment [36]. Others suggest that an association remains even after consideration of such factors and instead propose that low socioeconomic status acts as an independent risk factor for HIV mortality [38].

Unemployment contributes towards the perceived barriers to health care access [40], and there is reduced utilisation of health care services by the unemployed compared to their employed counterparts [41]. Whether employment status impacts upon HIV mortality through delayed health care access [39] or is an independent risk factor is currently unclear [25]. Our study does, however, confirm this association across a global data set.

The influence of unemployment on impaired mental well-being [14, 16] and increased suicidal tendencies has been well described [11, 18, 19]. The psychological sequelae associated with unemployment may also contribute towards increased HIV mortality.

Regarding PSEH, mechanisms are likely to focus on the availability of health care resources, which may be reduced during times of decreased PSEH. The era of highly active antiretroviral therapy (HAART) has seen vast improvements in HIV survival [42]. However, despite a gradual reduction in price, HAART remains an expensive therapeutic intervention. Importantly, better treatment outcomes of HAART are associated with the provision of free medication [43, 44]. It has also been shown that ineffective prophylaxis and treatment of co–morbidities, such as tuberculosis or opportunistic infections, can also contribute to higher HIV–mortality in low–income countries [44]. As a result both HIV–specific and general PSEH can have a direct impact upon HIV mortality.

It is likely that different mechanisms predominate in high–income and low–income settings. In high–income settings, the state tends to contribute towards the great majority to health care provision via PSEH, during times of recession there is also reduced long–term growth in private health insurance and out–of–pocket expenditure [7]. In lower–income countries, the contribution of the state is comparatively small and in the context of minimal private insurance cover, health care is funded primarily by out–of–pocket expenses [45]. As a result, economic crises can be particularly detrimental to health care access in such countries.

### Limitations

The introduction of bias was minimised from this study by only using data from high–quality, objective, centralised databases. Sufficient data was collected to allow us to capture multinational associative trends.

We recognise, however, that there are potential limitations to our study. Our evaluation of annual national data would have limited our ability to capture variations at the subnational level or within intra–year timeframes. HIV mortality served as the endpoint of our study; as a result we will have overlooked the influence of unemployment and PSEH on other health measures. We were unable to stratify our study by socioeconomic class – a factor which is known to have a significant influence on health care outcomes [36-39]. While we show a statistical association between unemployment and PSEH with HIV mortality, a causal link cannot be established. While we did intend for our study to have a truly global scope, a number of countries were omitted due to inadequate data for HIV mortality, unemployment and PSEH. In particular, only two countries in sub–Saharan Africa (Mauritius and South Africa) were included in our analysis despite the disproportionate burden of HIV in this region of the world. We also recognise that unemployment may not serve as an accurate barometer of individual financial well–being in less–developed countries. The poorest sub–strata within these populations may engage in work within the informal sector or in small–scale agriculture on subsistence farms. In such settings, government census data on employment status may be less meaningful. Our study is a retrospective, observational ecological study and so lacks the reliability of a prospective, experimental study. It may be subject to the influence of unknown or inadequately controlled confounders and cannot give strong evidence for causal attribution. Additional considerations, such as, indicators of political changes, occurrence of conflict or war, educational levels, and others, may permit any underlying mechanisms to be better determined. Further statistical techniques, such as interaction analyses could also provide insight into specific causal mechanisms. However, we believe this to be outside the immediate scope of this study which aimed to determine whether associations existed between the investigated variables.

Nevertheless, our study does establish an association between unemployment and PSEH with HIV mortality, thereby enabling a discussion of the trend on a supranational setting.

### Implications

Our study suggests that macro–level multinational policy could potentially impact upon mortality at the level of the individual, affecting day–to–day clinical practice. Times of reduced government spending and increased unemployment are likely to have worsened HIV mortality. It is possible that recently implemented austerity measures which have been associated with such changes are exacerbating the adverse health effects of the global economic downturn rather than ameliorating them.

In the current environment, policies that act to promote return–to–work or which prevent further unemployment could have tangible benefits in terms of HIV survival. Previous retrospective OECD analyses have shown that certain factors, such as employment protection legislation and work–sharing programmes, can confer resilience against unemployment rises during times of economic hardship [5]. Such strategies that actively maintain aggregate employment levels may also serve to protect population health during future recessions.

Caution must be taken in debates concerning health care cost restrictions and budget restrictions. If cost reductions are not achieved as a result of improvements in efficiency, they may entail deterioration in the quality of care and in turn greater mortality. Given that increases in health care spending, at least in the immediate timeframe, are unlikely, maximization of health care value is necessary to maintain and improve upon current HIV outcomes [46, 47].

## CONCLUSIONS

Recent economic turmoil has resulted in increased unemployment and decreased PSEH in countries around the world, raising the question of how economic changes, both within and outside crises, impact population health. Our study has shown that unemployment rises, and falls in PSEH, between 1981 and 2009, have been significantly associated with prolonged worsened HIV mortality. Policy interventions and austerity measures which negatively influence employment and PSEH may present additional barriers to HIV management.
