# Supplementary material for: Unemployment, public–sector health care expenditure and HIV mortality: An analysis of 74 countries, 1981–2009
Source: J Glob Health. 2015 Feb 10;5(1):010403. doi: 10.7189/jogh.05.010403 (PMC4337148; doi:10.7189/jogh.05.010403)
Supplement: Online Supplementary Document [file jogh-05-010403-s001.pdf]

Online Supplementary Document

Maruthappu et al. Unemployment, public–sector healthcare expenditure and HIV mortality: An analysis of 74 countries, 1981–2009

J Glob Health 2015;5:010403

Table S1. Mortality data completeness, coverage and quality

Country	Completeness	Coverage	Quality
Albania	60	69	Low
Argentina	100	100	Low
Armenia	68	91	Low
Australia	100	100	High
Austria	100	100	Med
Azerbaijan	74	71	Med
Bahrain	100	90	Low
Belarus	100	98	Med
Belgium	100	100	Med
Bosnia and Herzegovina	100	88	Low
Brazil	84	79	Med
Bulgaria	100	100	Med
Canada	100	100	High
Chile	100	100	Med
Colombia	81	79	Med
Costa Rica	90	79	Med
Croatia	100	98	Med
Cuba	100	100	High
Cyprus	100	70	Low
Czech Republic	100	100	Med
Denmark	100	100	Med
Ecuador	70	76	Low
Egypt, Arab Rep.	85	80	Low
El Salvador	76	73	Med
Estonia	100	100	High
Finland	100	100	High
France	100	100	Med
Georgia	75	66	Med
Germany	100	100	Med
Greece	100	88	Low
Guatemala	87	86	Med
Hong Kong SAR, China	N/A	N/A	N/A
Hungary	100	100	High
Ireland	100	100	High

Israel	100	100	Med
Italy	100	99	Med
Japan	100	97	High
Kazakhstan	89	80	Med
Korea, Rep.	89	88	Med
Kuwait	100	96	Med
Kyrgyz Republic	84	73	Med
Latvia	100	100	High
Lithuania	100	97	High
Macedonia, FYR	100	98	Med
Mauritius	100	100	Med
Mexico	100	96	High
Moldova	100	83	High
Montenegro	100	97	Med
Netherlands	100	100	Med
New Zealand	100	100	High
Norway	100	98	Med
Oman	N/A	71	Low
Panama	88	86	Med
Paraguay	74	74	Low
Philippines	77	85	Med
Poland	100	100	Low
Portugal	100	100	Low
Puerto Rico	N/A	N/A	N/A
Qatar	100	83	Low
Romania	100	100	High
Russian Federation	100	100	Med
Serbia	100	97	Med
Singapore	100	81	High
Slovak Republic	100	100	High
Slovenia	100	100	High
South Africa	88	<50	Low
Spain	100	100	Med
Sri Lanka	91	74	Low
Sweden	100	100	Med
Switzerland	100	100	Med
Thailand	86	89	Low
Trinidad and Tobago	100	93	Med
Ukraine	100	95	Med
United Kingdom	100	100	High
United States	100	100	High
Uzbekistan	100	100	Med
Uruguay	91	82	Med
Venezuela, RB	96	97	High

Table S2. Control variable coefficients

The coefficients of the control variables used in the PSEH analysis are included below

Variable	Both sexes	Male	Female
Population structure	-0.0000	-0.0000	-0.0000
Proportion of population >65 years	0.0192	0.0033	0.0246
Proportion of population <15 years	-0.0711	-0.0994	-0.0560

The coefficients of the control variables used in the unemployment analysis are included below

Variable	Both sexes	Male	Female
Population structure	0.0000	-0.0000	0.0000
Proportion of population >65 years	-0.0266	-0.0343	-0.0078
Proportion of population <15 years	-0.1806	-0.2622	-0.0965
